# PodCountNet: UAV-Based Soybean Pod Detection and Counting for High-Throughput Sink Trait Phenotyping

**DOI:** 10.3390/plants15213470

**Published:** 2026-11-06

**Authors:** Chang’An Zhou, Chenxiao Wang, Xiumin Guan, Shanxing Zhao, Jiarui Yang, Laichen Zheng, Jing Zhang, Xiaoying Ning, Keyan Peng, Jiutao Yang, Yuan Yao, Lanping Luo, Xin Liu

**Affiliations:** 1 Daizong Campus, Shandong Agricultural University, Tai’an 271018, China; zhouchangan@sdau.edu.cn (C.Z.); 2024120813@sdau.edu.cn (C.W.); 18764162569@163.com (J.Y.); 17866702112@163.com (L.Z.); 2024120802@sdau.edu.cn (X.N.); 2 Shandong Provincial Agricultural Technology Extension Center, Jinan 250100, China; greenmean@126.com (X.G.); 13854100052@163.com (K.P.); yangjiutao@shandong.cn (J.Y.); 19853139689@163.com (Y.Y.); lpluo8699@163.com (L.L.); 3 Zhaoxian Wucai Modern Agriculture Development Co., Ltd., Rizhao 276526, China; zsxab@sina.com; 4 Rural Economic Research Center, Ministry of Agriculture and Rural Affairs of the People’s Republic of China, Beijing 100810, China; 13811127087@126.com

**Keywords:** soybean, source–sink relationship, defoliation, photosynthesis, high-throughput phenotyping, UAV, pod counting

## Abstract

Photosynthetic source strength is a major determinant of soybean yield formation through its regulation of reproductive sink development. However, high-throughput quantification of sink traits under contrasting photosynthetic source conditions remains a major bottleneck for field-based source–sink physiology research. In this study, three soybean cultivars were subjected to graded artificial defoliation (0%, 25%, and 50%) at the R2 flowering stage to establish different levels of defoliation-induced source limitation. Biological observations showed that mature pod number consistently declined with increasing defoliation intensity across all cultivars, supporting the critical role of flowering-stage source limitation in determining sink formation. To enable efficient quantification of these sink responses, Unmanned Aerial Vehicle (UAV)videos acquired at maturity were used to construct a field soybean pod dataset, and an integrated detection–tracking framework, PodCountNet, was developed by combining an improved SPDM-YOLO detector with the OC-SORT tracking algorithm. The proposed SPDM-YOLO achieved a precision of 88.2% and an mAP@0.5 of 94.5% under diverse canopy conditions. Although single-view counting accuracy varied with canopy architecture and illumination, dual-view fusion effectively reduced systematic errors and, achieving MAE = 66.3 pods and RMSE = 88.0 pods at the individual plot level (*n* = 27), substantially outperforming single-view front-lit (MAE = 114.2) and back-lit counting (MAE = 172.9). The proposed framework enables accurate, non-destructive, and high-throughput quantification of soybean sink responses under contrasting photosynthetic source conditions, providing an effective phenotyping tool for investigating source–sink coordination and supporting photosynthesis-oriented soybean breeding.

## 1. Introduction

Photosynthesis supplies over 90% of the dry matter accumulated in crop plants and constitutes the primary physiological basis of crop yield formation [1,2]. In soybean, reproductive development and final grain yield depend heavily on the balance between source strength—the capacity of leaves to produce photosynthetic assimilates—and sink demand from reproductive organs [3,4]. Among yield components, pod number is the most representative sink trait, as it directly reflects the efficiency of assimilate partitioning from source leaves to reproductive structures during critical growth stages [5,6]. Any reduction in photosynthetic leaf area restricts assimilate availability, alters canopy light interception, and consequently influences pod set, pod retention, and final yield [3,5,7]. Understanding how photosynthetic source variation shapes pod development is therefore central to breeding for enhanced photosynthetic efficiency and yield improvement.

Artificial defoliation has long been established as a robust experimental approach to manipulate canopy photosynthetic capacity and investigate source–sink relationships in soybean [5,7,8]. For vegetative growth stages, defoliation at the V3 seedling stage has been extensively characterized. For example, Cai et al. demonstrated that V3-stage defoliation reduced soybean yield in a dose-dependent manner, which was mainly mediated by decreased leaf area index, suppressed dry matter accumulation, and altered vertical pod distribution [7]. However, the R2 (full flowering) stage—the critical transition window from flower development to pod initiation—has received far less systematic investigation. Assimilate supply at this stage directly determines flower retention rate and the number of successfully initiated pods, and further shapes canopy architectural traits at maturity. The impacts of R2 source limitation on final pod number, and on the performance of field phenotyping methods across the resulting diverse canopy phenotypes, remain poorly characterized.

In this study, we selected the R2 full-flowering stage for defoliation treatments to capture the early reproductive window when source strength first influences pod set. By imposing graded levels of leaf removal at R2, we simulated reductions in source strength during this sensitive period, altered subsequent canopy architecture and light distribution, and quantified impacts on final pod number at maturity. Such defoliation gradients generate highly diverse canopy phenotypes with varying degrees of pod occlusion, illumination heterogeneity, and background complexity. However, phenotyping pod number across such diverse canopy conditions remains a major bottleneck because conventional manual counting is destructive, low-throughput, and impractical for large-scale source–sink field experiments [9,10,11].

Current field-scale evaluations of photosynthesis-related yield performance rely predominantly on UAV-based multispectral or hyperspectral remote sensing, which estimates yield through vegetation indices or biomass proxies [12,13,14]. While these methods operate at high throughput, they cannot directly quantify sink traits such as pod number [3,4]. The inability to directly quantify pod number at field scale means that researchers cannot measure the most important sink trait in source–sink studies, forcing reliance on indirect yield proxies that cannot distinguish whether yield changes arise from pod number, seeds per pod, or seed weight. This gap motivates the development of a direct, non-destructive pod counting method suitable for field-scale source–sink experiments. Computer vision and deep learning provide a promising route toward non-destructive pod quantification, but most existing soybean pod detection models have been developed under relatively controlled environments [9,10,11,15]. When transferred to complex field canopies generated by different defoliation intensities, performance often declines because of dense pod distribution, severe occlusion and variable illumination. Furthermore, most studies focus on single-image detection, whereas continuous video-based pod counting remains largely unexplored [9,10,15].

To address these gaps, this study proposes PodCountNet, an integrated detection–tracking–counting framework for high-throughput soybean pod phenotyping under variable photosynthetic source conditions. The main contributions are as follows:

(1) An improved SPDM-YOLO model was developed based on YOLOv11n, incorporating LSKNet for dynamic receptive field adjustment, FDPN for enhanced small-target feature extraction, and TADDH for decoupled classification and regression. The model achieves robust pod detection across diverse canopy structures generated by different defoliation intensities.

(2) The OC-SORT multi-object tracking algorithm was integrated with SPDM-YOLO to enable continuous video-based pod counting. Kalman filtering and the Hungarian algorithm were employed for state prediction and data association, reducing repeated counting and missed detection caused by UAV movement and pod occlusion under variable canopies.

(3) A dual-view (front-lit and back-lit) data fusion strategy was proposed to mitigate counting errors caused by illumination variation across canopy architectures. By fusing counting results from two perspectives via binary quadratic polynomial regression, the framework achieved MAE = 66.3 pods and RMSE = 88.0 pods at the individual plot level, substantially reducing error compared with single-view counting.

Overall, the proposed PodCountNet framework extends soybean pod phenotyping from static indoor image analysis to continuous UAV video-based field phenotyping. It provides a practical high-throughput tool for quantifying sink trait responses to photosynthetic source variation, and supports research on source–sink coordination and photosynthesis-based yield improvement in soybean.

## 2. Materials and Methods

### 2.1. Experimental Site

The experimental study area is located at the Wenyangtian Modern Agricultural Industrial Park experimental field in Feicheng City, Tai’an City, Shandong Province. This base is situated on the western foothills of the central Shandong Mountains (approximately 36°12′15.60” to 36°13′02.30” N, 116°44′38.50” to 116°45′17.80” E), with an average elevation of approximately 65 m. We selected a 4263 m^2^ plot as our experimental field.

This region features a warm temperate, semi-humid continental monsoon climate with distinct seasonal changes and an annual average precipitation of 630–710 mm. The predominant soil type is brown earth, characterized by deep soil layers with organic matter content in the topsoil ranging from 2.8% to 4.5% and a pH level of 6.5–7.2, offering excellent water and nutrient retention properties. The agricultural system employs a double-cropping system, with climatic conditions suitable for soybean cultivation. The area also benefits from convenient transportation, facilitating experimental operations such as UAV aerial photography.

**Figure 1:**
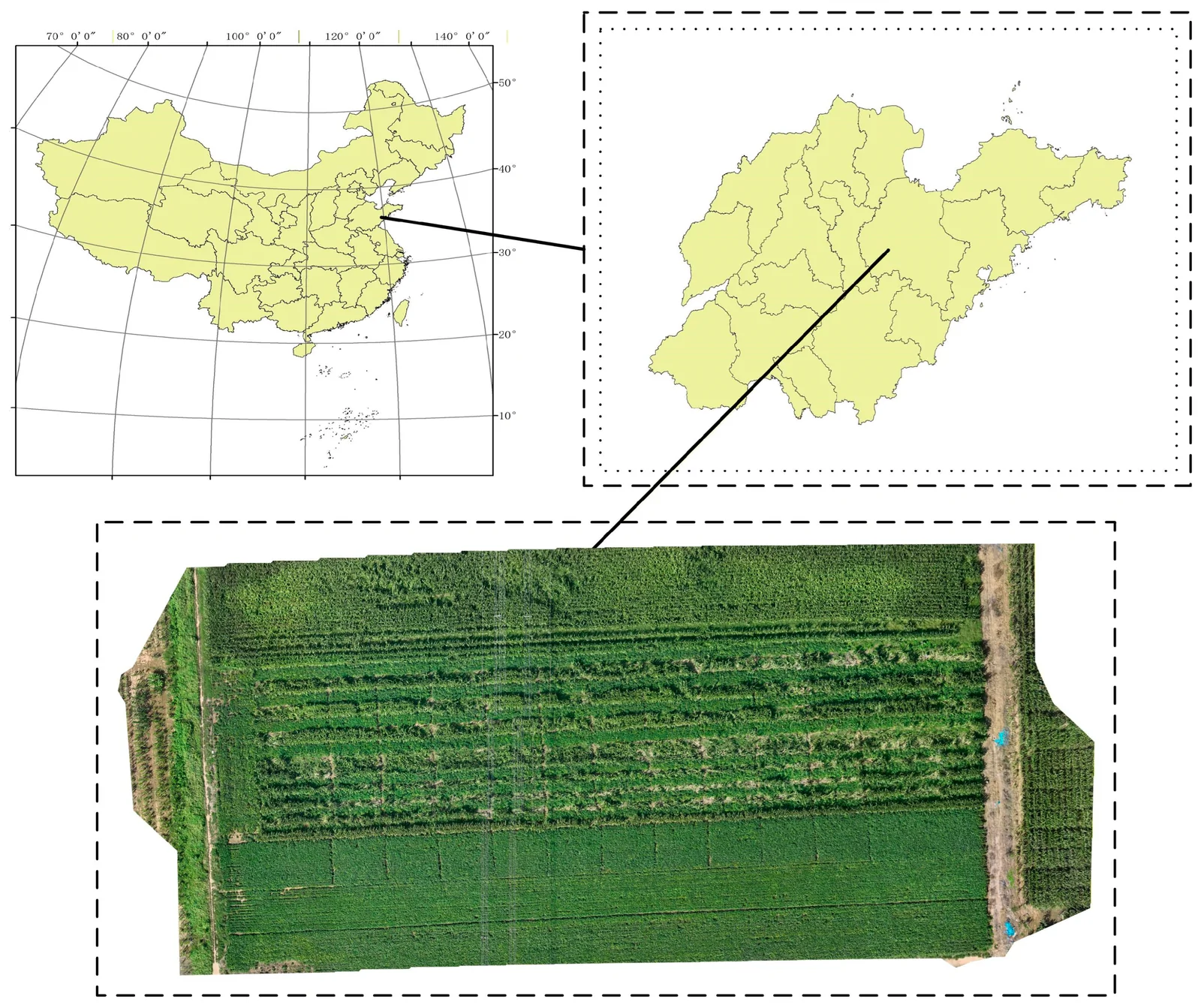
Overview of the study area.

**Figure 2:**
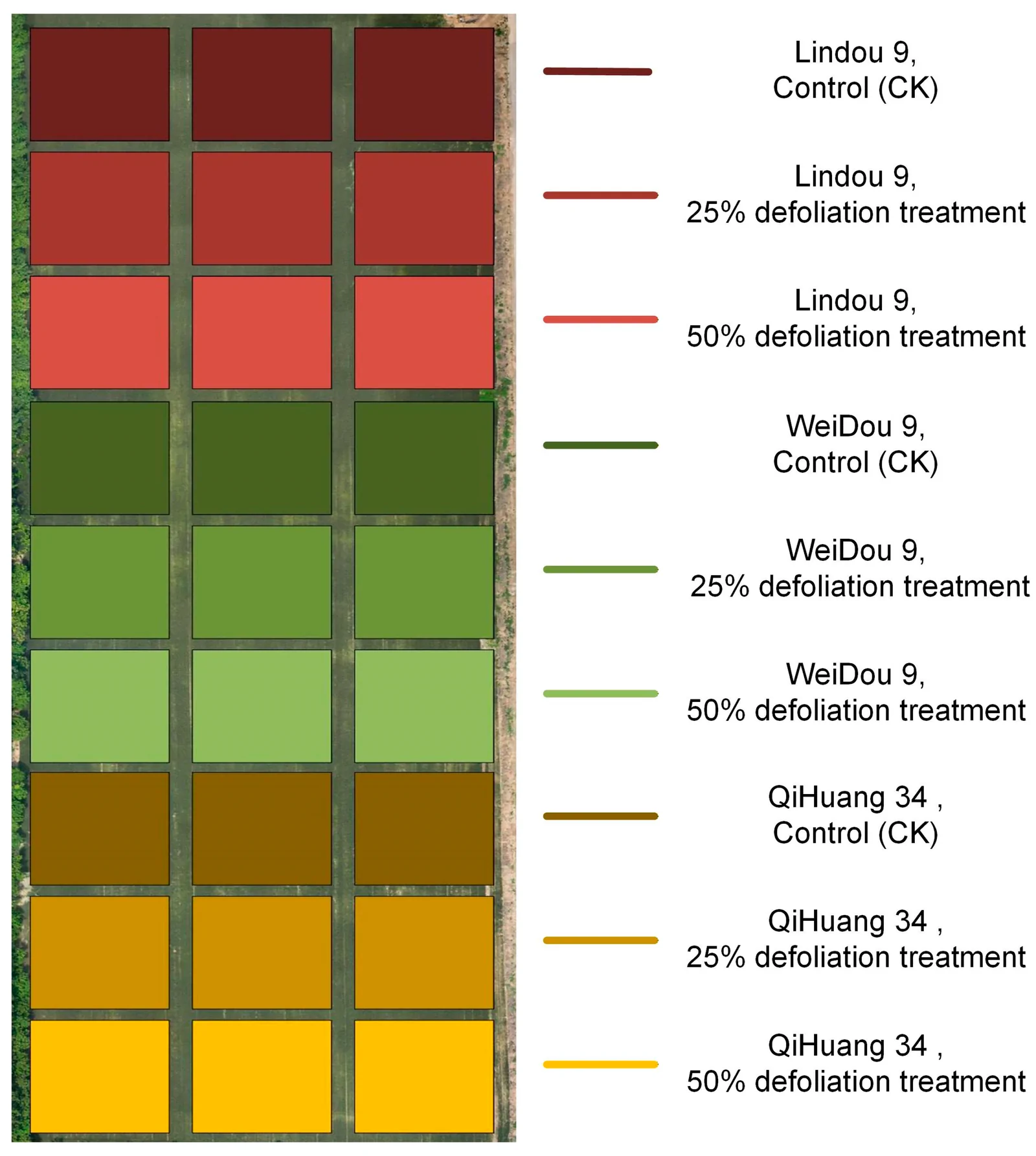
Field Study Site Configuration. The three soybean cultivars (Lindou 9, Weidou 9, and Qihuang 34) and three defoliation treatments (CK, 25%, 50%) are labeled in the field layout.

Soybean varieties Qihuang 34, Lindou 9, and Weidou 9 were selected for planting. These three cultivars differ in pod morphology, plant architecture, and yield potential, and are referred to by these names throughout the manuscript and in all figure labels. Sowing occurred on 7 June 2024, with harvest on 13 October 2024. Field management measures, including irrigation and pesticide spraying, were implemented to protect the soybeans from drought, pests, diseases, and weeds. To construct a high-information phenotypic data training set, a split-plot design was adopted. The main plots represented the varieties, while the defoliation treatments served as subplots. Plots were randomly arranged in three replicates, totaling 27 plots. The study area location and field study site configuration are shown in Figure 1 and Figure 2, respectively.

The defoliation treatments were applied at the R2 (full flowering) growth stage, which occurs approximately 5–6 weeks after sowing and represents the early reproductive window when assimilate supply determines flower retention and pod initiation. Three defoliation levels were set: no defoliation (CK), 25% leaf removal, and 50% leaf removal. Defoliation was performed on 30 July 2024, whereas UAV video acquisition was conducted on 5 October 2024, at the R8 (full maturity) stage—approximately 9–12 weeks after defoliation. This temporal separation ensured that the pod number quantified by UAV imaging reflected the cumulative biological outcome of source limitation during flowering (i.e., flower abortion, pod set, and pod retention), rather than immediate physical obstruction by removed leaves. By the maturity stage, all leaves had naturally abscised across all treatments, so canopy differences among plots consisted solely of stems, branches, and pods.

For both defoliation treatments, all leaf removal was performed manually by trained field workers, and unified systematic removal protocol was adopted to ensure uniform leaf loss along the canopy. Specifically, the removal interval was determined by the target defoliation percentage: in the 25% treatment, one out of every four leaves was removed (i.e., three leaves retained followed by one removed); in the 50% treatment, one out of every two leaves was removed (i.e., one leaf retained followed by one removed). Counting started from the uppermost fully expanded compound leaf and proceeded downward, and the same retain–remove cycle was repeated along the entire plant. Only the leaf blades were detached, with petioles retained to avoid stem damage. This approach ensured that the predetermined defoliation intensity was achieved precisely while distributing the leaf loss evenly across upper, middle, and lower canopy layers.

All defoliation operations were conducted on sunny mornings to minimize plant stress and wound infection risk. Each treatment was replicated three times across the split-plot design.

It should be noted that these defoliation treatments imposed graded levels of source limitation without directly quantifying the corresponding reduction in photosynthetic capacity, leaf area index (LAI), or photosynthetic rate; therefore, the exact magnitude of photosynthetic reduction associated with each defoliation percentage was not measured in this study.

### 2.2. Acquisition of UAV Video Data

Data collection took place on 5 October 2024, utilizing a DJI Mini 4 Pro UAV for manual ultra-low-altitude flight to perform image acquisition tasks. The DJI Mini 4 Pro was selected for its compact size and light weight, which produces minimal rotor downwash at the 1-m flight altitude. Moreover, data acquisition was conducted at the maturity stage (R8), when most leaves had abscised and pod moisture content was low, making the canopy less susceptible to wind-induced sway. A UAV-based platform was also preferred to avoid soil compaction and plant damage caused by ground-based vehicles in narrow-row field layouts. Flying at a height of one meter, the UAV’s camera was set at a 45° downward angle to capture high-resolution, detailed plant images. Each 6-m plot corresponded to different combinations of plant varieties and defoliation treatments, and the general layout of the study area and trial plots is shown in Figure 2. Two video segments were recorded per plot (one in front-lit and one in back-lit conditions). Front-lit and back-lit conditions were achieved by adjusting the UAV heading direction relative to the sun’s azimuth: in front-lit flights the camera faced toward the sun-illuminated side of the canopy, whereas in back-lit flights the camera faced away from the sun, placing the canopy between the sun and the sensor. Because each 6-m plot was traversed in approximately 12 s at 0.5 m/s, solar elevation and illumination conditions remained effectively constant within a single video segment, capturing diverse data across multiple varieties, treatments, and lighting conditions for subsequent analysis. To ensure spatial consistency between the front-lit and back-lit flights of the same plot, both flights were initiated from the same marked ground reference point at the southern end of each plot, followed the same soybean row northward, and maintained the same 1-m altitude and 0.5 m/s flight speed. The pilot used fixed ground markers at both ends of each plot for visual alignment. During data collection, the flight speed of the UAV was fixed at 0.5 m/s. The DJI Mini 4 Pro UAV was used with its default video shooting settings: 4K resolution at 30 fps, 1/1.3-inch CMOS sensor, equivalent 24 mm focal length, f/1.7 aperture, and 3-axis mechanical gimbal stabilization. The ISO range was set to 100–3200, and the electronic shutter was used with automatic adjustment according to lighting conditions. All data were collected between 10:00 and 16:00 Beijing time on sunny days. In addition to the 27 experimental plots, extra flight surveys were conducted under the same flight conditions over adjacent soybean rows of the same three cultivars and defoliation treatments to provide raw video footage for dataset construction.

Following UAV data acquisition, all 27 plots were manually harvested on 13 October 2024, and the total pod number along each 6-m flight trajectory was counted manually by trained field workers. These manual counts served as the ground-truth reference values for evaluating the accuracy of the tracking-based pod counting method. Each plot yielded one front-lit video, one back-lit video, and one corresponding manual ground-truth pod count. The experiment employed a split-plot design with three cultivars (main plots), three defoliation treatments (subplots), and three biological replicates, totaling 27 plots.

### 2.3. Dataset Construction

This study utilizes soybean video footage acquired from the additional flight surveys described in Section 2.2 as its data source. The videos contain images of soybean plants that are clear, blurred, and varying degrees of obscured, fully reflecting the diversity of actual cultivation scenarios [16,17]. First, non-overlapping RGB frames with a resolution of 3840 × 2160 pixels were extracted from the videos, with the sampling interval adjusted to ensure that no individual plant appeared in more than one frame. Subsequently, each extracted frame was cropped without overlap to obtain 1296 × 768 pixel sub-images. This dimension selection is primarily based on two considerations: first, it significantly reduces computational resource consumption, enabling efficient processing of large-scale datasets while accelerating model training and inference speeds [18]; Second, improve the speed and accuracy of initial manual annotation [19,20]. To construct a high-quality dataset with limited annotation resources, this study employs an active learning strategy. First, 200 representative images covering all categories are precisely annotated manually using LabelImg from the entire dataset, forming the initial training set to train a preliminary SPDM-YOLO detection model. Subsequently, this model is used to infer the remaining images, identifying high-value samples with high classification uncertainty, ambiguous boundaries, or complex backgrounds. After professional review and annotation, these samples are added to the training set. Through repeated iterations of the “inference–selection–annotation–training” process, both model performance and data quality are progressively enhanced, ultimately forming a dataset comprising 1443 images.

**Figure 3:**
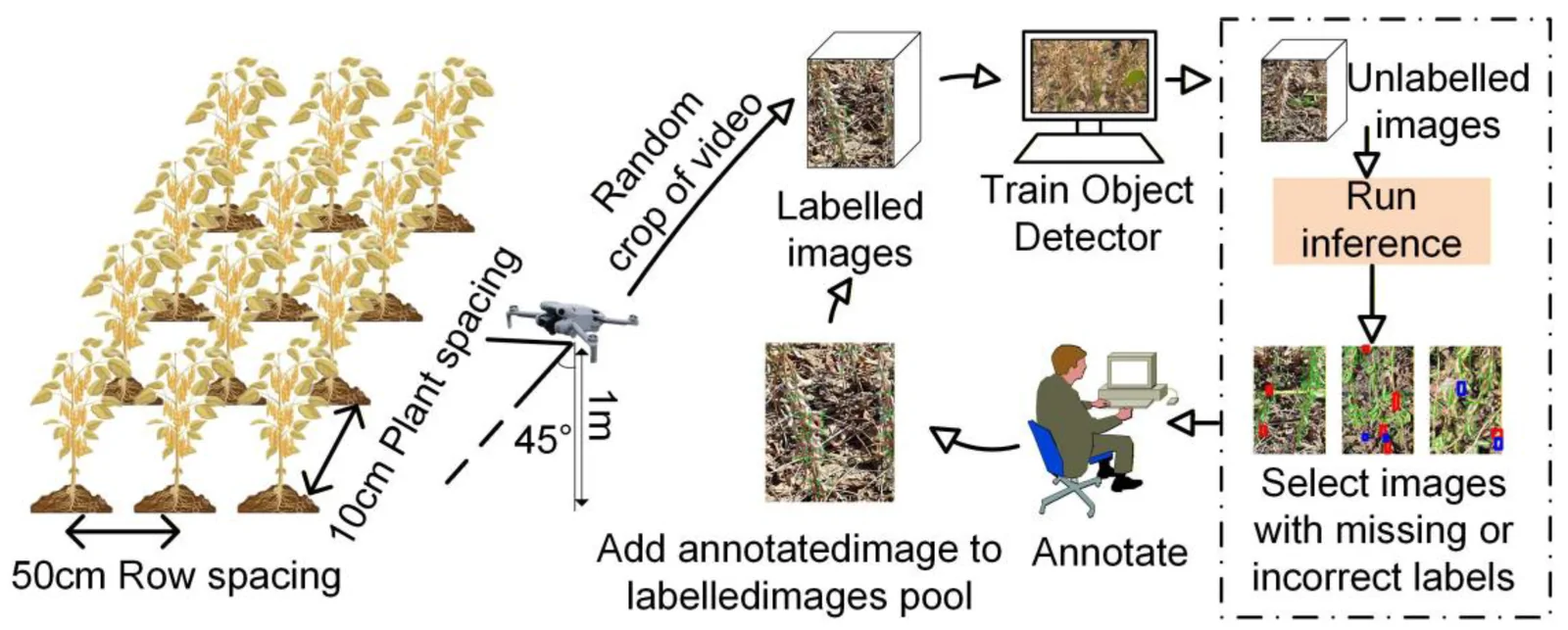
Method for constructing the research dataset.

The dataset was ultimately split into training, validation, and test sets in a 7:2:1 ratio [21,22]. Because frames were non-overlapping at the plant level, each split contained distinct plant individuals with no cross-set redundancy. Through the adoption of active learning, a high-quality dataset representing complex field environments was constructed with only partial sample annotation. This strategy significantly boosted labeling efficiency and yielded a reliable data basis for follow-up high-precision detection. Images were annotated in YOLO format using LabelImg software (version 1.8.6) by three professionally trained annotators independently. After the annotation work was fully completed, all annotation results were rechecked, revised and finalized by agronomy experts to ensure the accuracy and biological rationality of the annotations. The method for constructing the research dataset is illustrated in Figure 3.

### 2.4. PodCountNet Framework Construction

#### 2.4.1. YOLOv11

The PodCountNet model developed in this study is built upon the YOLOv11n framework as its foundation. Compared to previous versions in the YOLO series, the YOLOv11n object detection model introduces several architectural innovations, which further enhance its detection performance and operational flexibility [23]. The network architecture of the proposed model is primarily composed of three components: the Backbone, Neck, and Head. Within this framework, the Backbone is responsible for extracting target features from input images, aiming to distill embedded visual information for subsequent processing by other network components. The Neck, situated between the Backbone and the Head, facilitates enhanced integration and refinement of the features derived from the Backbone. Finally, the Head serves as the output module of the detection network, leveraging the aggregated features to predict both the class and spatial location of targets within the image. Given the small size of soybean pods and their visual similarity to the plant background during the harvest period, the detection network must prioritize improved accuracy to ensure reliable performance [24].

#### 2.4.2. Architectural Design of SPDM-YOLO

**Figure 4:**
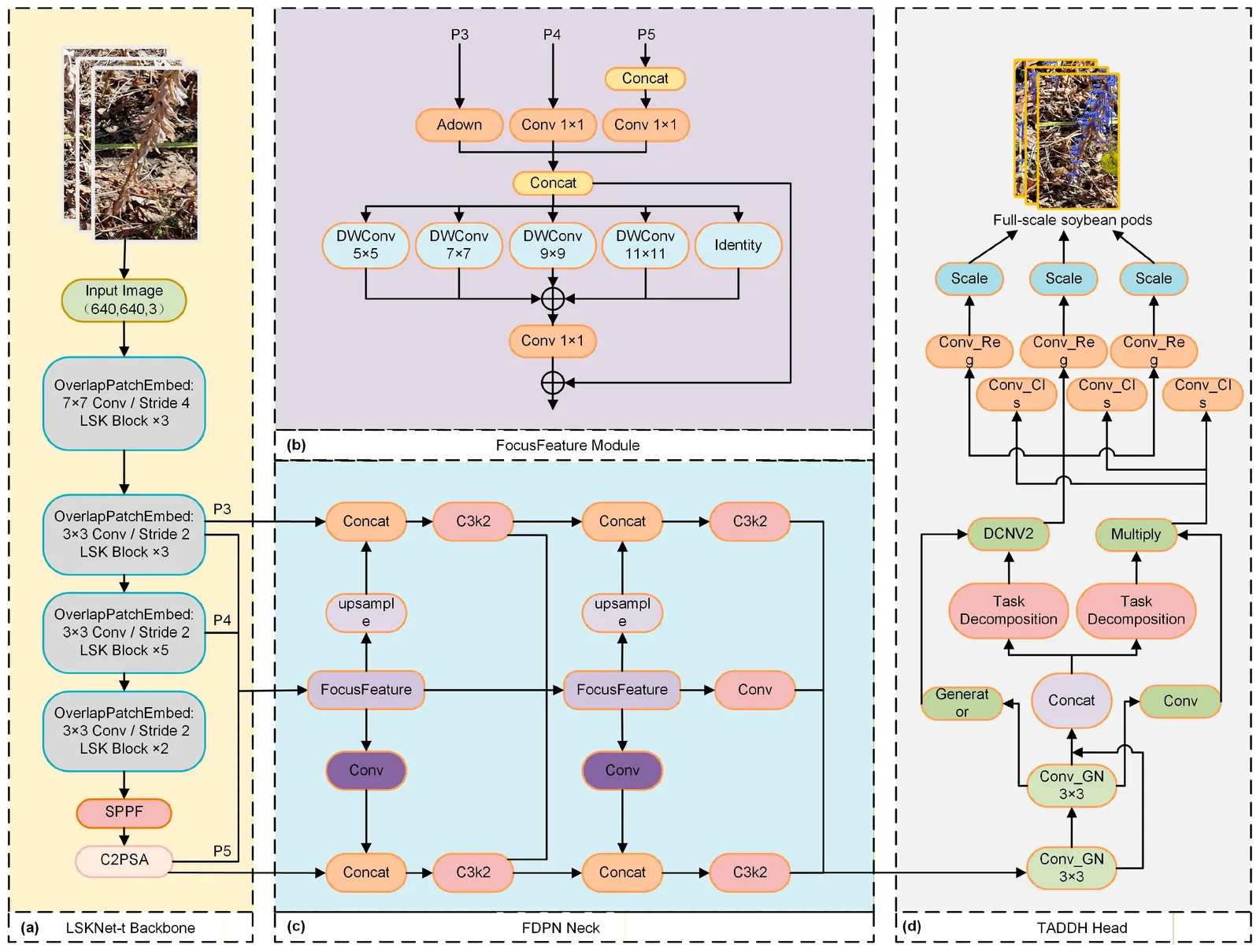
SPDM-YOLO model architecture for soybean pod detection. (**a**) LSKNet-t Backbone; (**b**) FocusFeature module; (**c**) FDPN neck; (**d**) TADDH head.

Three core improvements were implemented to address the key characteristics of soybean pods: their small size, high density, and the complexity of their background. The proposed soybean pod detection network is built upon YOLOv11n. Its backbone incorporates LSKNet [25], which dynamically adjusts the receptive field to handle scale variation. To further enhance performance, the neck network is replaced with a Feature-focused Diffusion Pyramid Network (FDPN) [26]. This architecture reduces redundant information through feature decoupling and cross-layer fusion, thereby strengthening pod detection capability. Moreover, the original YOLOv11n detection head is substituted with an Aligned Dynamic Detection Head (TADDH) [27], which improves detection accuracy, especially in scenarios with dense targets and small objects. Collectively, these enhancements result in a more efficient and accurate model for soybean pod detection, recognition, and counting. The improved model effectively advances the localization and classification of soybean pods, demonstrating particularly strong performance in small-object detection tasks. The architecture of the refined soybean pod detection network is presented in Figure 4.

#### 2.4.3. LSKNet

**Figure 5:**
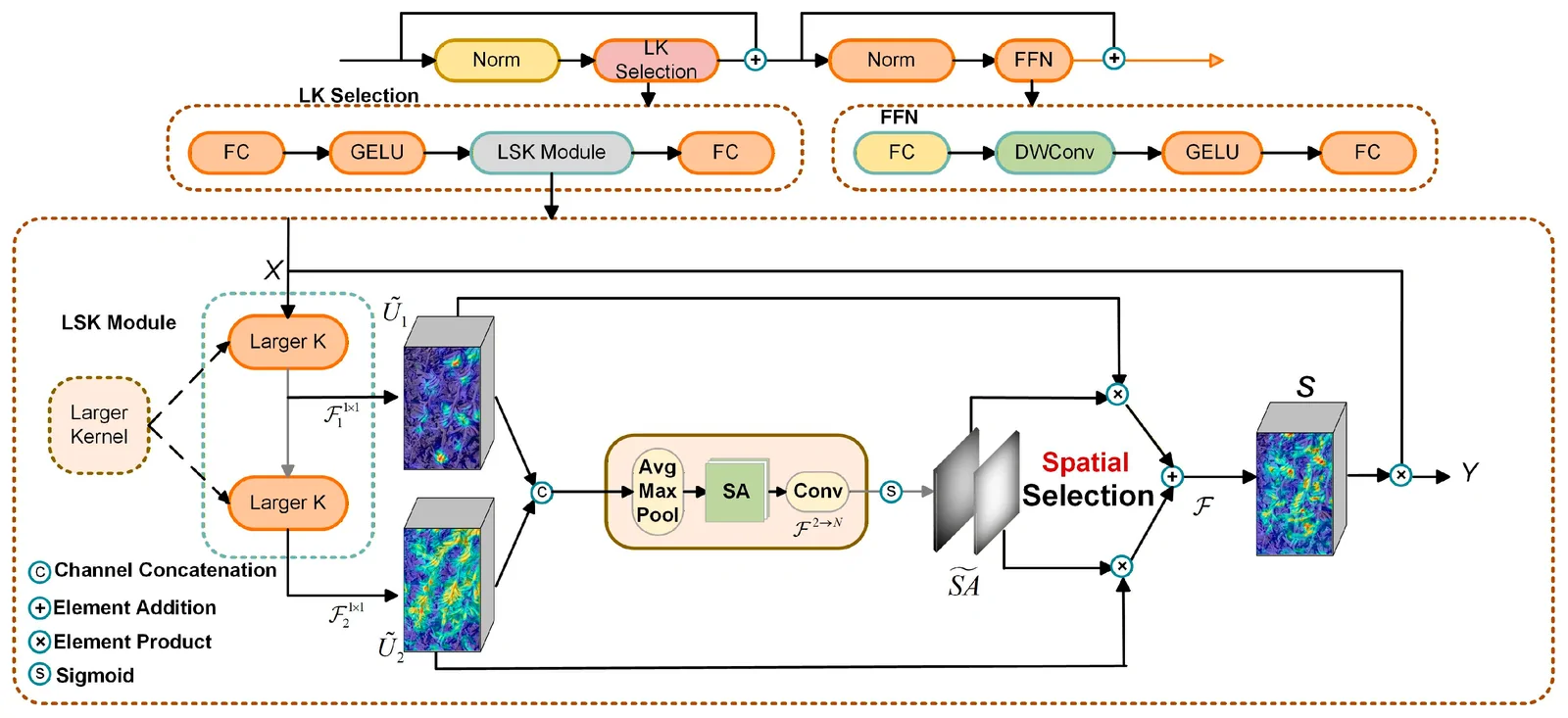
LSK Block.

In the backbone network section, this study employs the LSKNet model optimized for detecting small targets in remote sensing. The perspective distortion issue—where pods appear larger at close range and smaller at distance—in drone-captured videos results in significant size variations among pods in the images. LSKNet employs a sequence of convolutional, pooling, and residual modules, combined with multi-scale feature fusion, to generate high-resolution feature maps covering different receptive field ranges. This effectively enhances the representation capability for pod targets at various scales. The LSKNet block comprises an LK Selection sub-block and a feed-forward network (FFN) sub-block. The LK Selection sub-block dynamically adjusts the network’s receptive field, while the FFN sub-block performs channel mixing and feature refinement. It consists of one fully connected layer, one deep convolutional layer, a GELU activation function, and two fully connected layers. The LSK module is embedded within the LK Selection sub-block, with its structural composition illustrated in Figure 5.

#### 2.4.4. FDPN (Focusing Diffusion Pyramid Network)

Traditional FPN algorithms exhibit high rates of false positives and false negatives when detecting small objects against complex backgrounds. This study replaces the original neck network in YOLOv11n with the Focusing Diffusion Pyramid Network (FDPN) to enhance the model’s detection capability for small objects like pods. FDPN improves feature fusion efficiency by employing a custom feature focusing module and diffusion mechanism, ensuring each scale of features possesses detailed contextual information. Building upon FPN, FDPN employs decoupling to process semantic and fine-grained features separately before dynamically fusing them. It decomposes feature maps from the backbone network (e.g., C3, C4, C5) into semantic and fine-grained components. Semantic pyramids are constructed through upsampling and convolutions, while fine-grained pyramids are generated via convolutions, batch normalization, and activation functions. A dynamic weight mechanism fuses features from the semantic and detail pyramids to generate final multi-scale feature maps (P3, P4, P5), enhancing expressive power across scales and demonstrating strong performance in detecting small objects (e.g., pods). However, this performance gain comes at the cost of increased parameter computation and computational demands. The structural diagram of the FDPN module is shown in Figure 4c.

#### 2.4.5. TADDH

Since the original detection head of YOLOv11n is a static multi-task detection head, the weights for each branch are fixed and cannot be dynamically adjusted based on the content of the input feature map. The classification, regression, and confidence branches share the same feature map, potentially causing interference between tasks. This leads to insufficient detection accuracy in complex scenes and poor detection performance for small and occluded objects. This study introduces the Aligned Dynamic Detection Head (TADDH) based on YOLOv11n’s original detection head. It aims to achieve more precise task alignment by designing a novel head architecture and an alignment-oriented learning method. First, shared convolutions are employed to significantly reduce the number of parameters, making the model lighter and compensating for the increased computational load after replacing the neck network. Additionally, to address inconsistent object scales detected by each detection head, a Scale layer is employed to resize features. TADDH learns task-interaction features from multiple convolutional layers via its feature extractor, generating joint features. Its localization branch uses DCNV2 and interaction features to produce DCNV2 offsets and masks, while the classification branch performs dynamic feature selection using interaction features. DCNV2 enhances deformable convolution applications by enabling flexible sampling across multi-layer features through multi-stage offset learning. Its modulation mechanism adjusts sampling point positions and strengths, allowing the network to dynamically optimize sampling layouts and influence weights for enhanced adaptability. This facilitates smoother collaboration between tasks, enabling more precise prediction adjustments. The TADDH detection head architecture is illustrated in Figure 4d.

#### 2.4.6. OC-SORT

**Figure 6:**
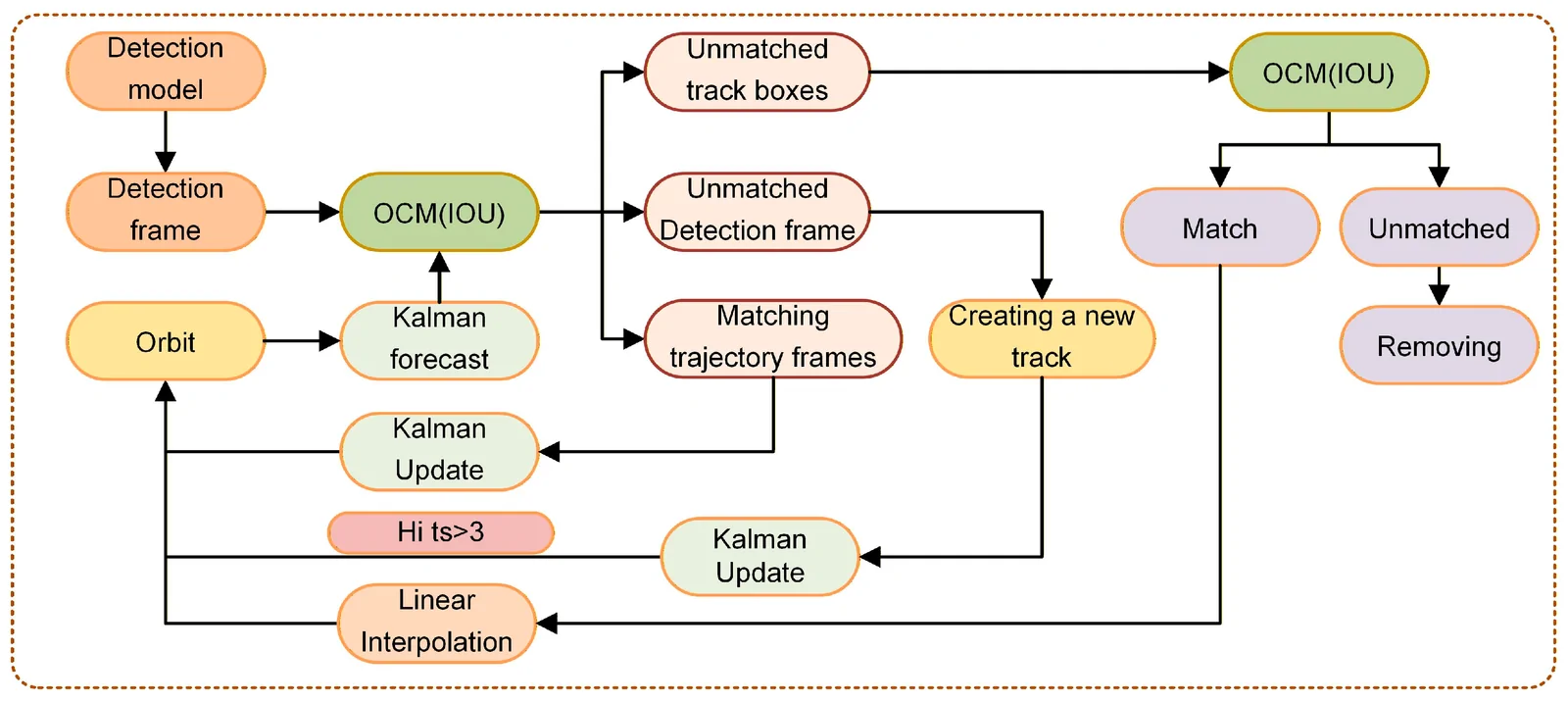
OC-SORT Tracking Algorithm.

Given the visual similarity between soybean pods and the field background, coupled with instances of overlap and occlusion involving soybean branches and leaves, this study introduces the OC-SORT (Observation-Centric SORT) object tracking algorithm to achieve accurate detection and recognition of drone-captured video data. The network architecture is shown in Figure 6. OC-SORT is an observation-based multi-object tracking algorithm optimized for complex scenarios involving small object recognition, such as target occlusion and non-linear motion [28]. During the detection phase, the trained SPDM-YOLO object detector was used to generate the soybean pod detection bounding boxes for each frame of the video. The Intersection over Union (IoU) or Mahalanobis distance between detection boxes and existing tracks is calculated. The Hungarian algorithm is employed for matching, prioritizing observation data over predicted states. New tracks are initialized for unmatched detection boxes. Lost tracks are temporarily retained to attempt recovery. Trajectories that remain unmatched for extended periods are discarded to prevent erroneous tracking. Trajectory states are updated using Kalman filtering, with observations taking precedence. The motion model is optimized by incorporating directional consistency constraints. Compared to SORT and DeepSORT, OC-SORT demonstrates greater robustness in scenarios involving occlusions and non-linear motion. Furthermore, its low computational complexity makes it well-suited for real-time applications [29]. In this study, the IOU matching threshold of OC-SORT was set to 0.3, the maximum retention frame number of lost tracks was 30, and a new track was officially generated only when the target was continuously detected in 3 frames to avoid false tracks caused by background interference.

#### 2.4.7. Video-Based Pod Counting Pipeline

The video counting pipeline employed a virtual-line counting strategy based on pod-level multi-object tracking. The SPDM-YOLO detector identified individual soybean pods in each frame, and the tracking algorithm (OC-SORT) assigned a unique and persistent trajectory ID to each detected pod via Kalman filtering state prediction and Hungarian algorithm data association. A vertical virtual counting line was placed at a fixed horizontal position in each frame. As the UAV moved along the soybean row at a constant speed, each pod’s trajectory was monitored across consecutive frames. When a pod’s center crossed the virtual counting line, its unique trajectory ID was registered and counted exactly once. Because each pod was associated with a single persistent ID throughout its visible lifetime, frame-to-frame position jitter or brief occlusion did not cause the same pod to be counted multiple times. This trajectory-based crossing-counting approach counts unique pod IDs rather than summing bounding boxes per frame, thereby avoiding duplicate counting. All frames of each video segment were processed sequentially without subsampling, and the final pod count for each plot was the total number of unique pod IDs that crossed the counting line. On an NVIDIA RTX 3080 GPU, the detection–tracking pipeline processed video at approximately 4 FPS inference speed at 1280 × 768 input resolution, enabling efficient offline processing for field phenotyping.

#### 2.4.8. Regression Model for Pod Count Prediction

Soybean pods in the video data were quantified using the SPDM-YOLO model coupled with the OC-SORT multi-object tracking algorithm, yielding video-based pod counts for each experimental plot. These automated counts were then integrated with corresponding manually measured pod quantities to construct a binary quadratic polynomial regression for pod number estimation.

Within the experimental field where the image data were acquired, each sampling plot was defined as a 6-m row. A uniform 6-m planting belt was designated as one plot, with consistent soybean planting density maintained across all plots.

### 2.5. Experimental Configuration and Evaluation Metrics

The experimental platform for model training and testing was equipped with an i9-12900K CPU, 32 GB of RAM running at 3200 MHz, and an NVIDIA GeForce RTX 3080 GPU. The training environment comprised Python 3.7, Anaconda3, the PyTorch 2.2.2 (CUDA 12.1) deep learning framework, Microsoft Windows 10 Professional (64-bit) as the operating system, and PyCharm Community Edition 2022.2.3 as the primary development and execution platform.

In this study, we selected precision (P), recall (R), and mean average precision (mAP) as the evaluation metrics for target detection. The formulas for P, R, and mAP are presented below.

(1) $$P=\frac{TP}{TP+FP}$$

(2) $$R=\frac{TP}{TP+FN}$$

(3) $$mAP=\frac{\sum precision}{Nt}$$

These metrics are defined based on the following terms: the number of correctly identified pods (TP), the number of objects incorrectly classified as pods (FP), the number of undetected pods (FN), the total number of samples (N), and the number of target categories detected (C).

For pod number estimation, the Mean Absolute Error (MAE) and Root Mean Square Error (RMSE) were adopted as the evaluation metrics for the proposed pod number estimation method. Additionally, the coefficient of determination (R^2^) was used to evaluate how well the model explains the variance in the observed yield data.

(4) $$R^{2}=1-\frac{\sum_{i=1}^{n}{\left(y_{i}-{\hat{y}}_{i}\right)}^{2}}{\sum_{i=1}^{n}{\left(y_{i}-\bar{y}\right)}^{2}}$$

(5) $$MAE=\frac{1}{n}\sum_{i=1}^{n}|y_{i}-{\hat{y}}_{i}|$$

(6) $$\mathrm{RMSE}=\sqrt{\frac{1}{n}\sum_{i=1}^{n}{\left(y_{i}-{\hat{y}}_{i}\right)}^{2}}$$

where $y_{i}$ is the actual observed value, ${\hat{y}}_{i}$ is the model-predicted value, and $\bar{y}$ is the mean of the observed values.

The pre-trained weights of yolov11n were loaded for transfer learning to accelerate model convergence. The key training parameters were set as follows: input image size (imgsz) of 640 × 640, total training epochs of 300, batch size of 16, SGD optimizer, and patience of 50 for early stopping (training stops if validation loss does not decrease for 50 consecutive epochs).

## 3. Results

### 3.1. Training Results of SPDM-YOLO

**Figure 7:**
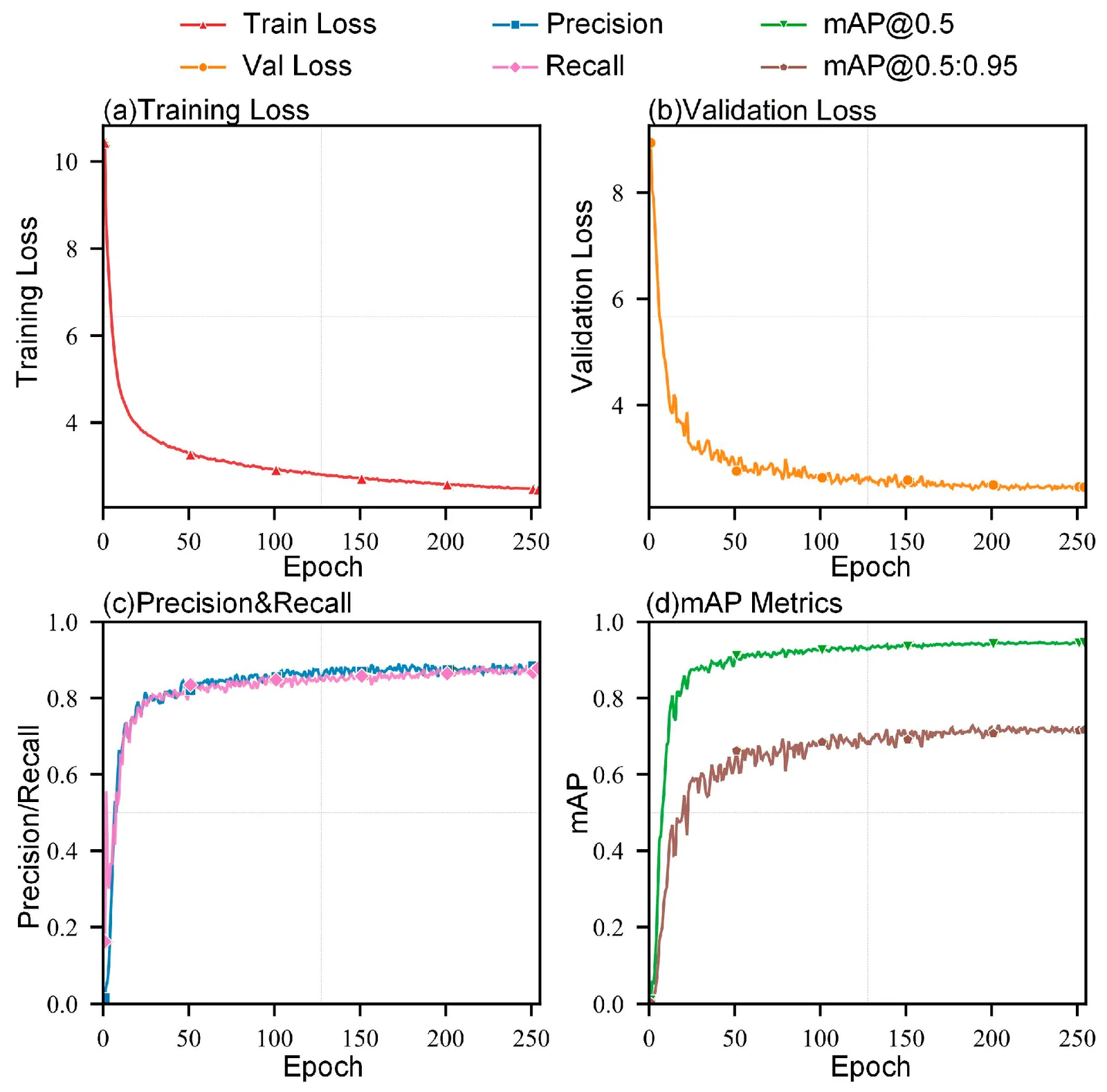
Training Results of SPDM-YOLO.

The training results of SPDM-YOLO on the dataset are shown in Figure 7. The model training employed an early stopping mechanism, with a total of 254 epochs completed. Notably, the loss curve stabilized within the first 50 epochs, indicating significant improvements in detection performance. As training progressed, precision, recall, and mAP@0.5 began stabilizing around the 100th epoch. The early stopping mechanism was triggered at the 254th epoch, as the validation loss did not decrease for 50 consecutive epochs from the 204th epoch. The final detection precision reached 88.2%, recall achieved 87.0%, and mAP@0.5 attained 94.5%. These results meet the performance requirements for pod detection in complex field environments.

### 3.2. Ablation Experiments

To validate the performance of the proposed SPDM-YOLO model, we conducted systematic ablation experiments for comparative evaluation. In this experimental procedure, we ablated each of the three enhancement modules individually and in pairwise combinations—LSKNet in the backbone, FDPN in the neck network, and TADDH in the detection head—while training and evaluating all variants using the same dataset. This process aims to quantify the individual and combined contributions of each enhancement mechanism to the pod detection model.

**Figure 8:**
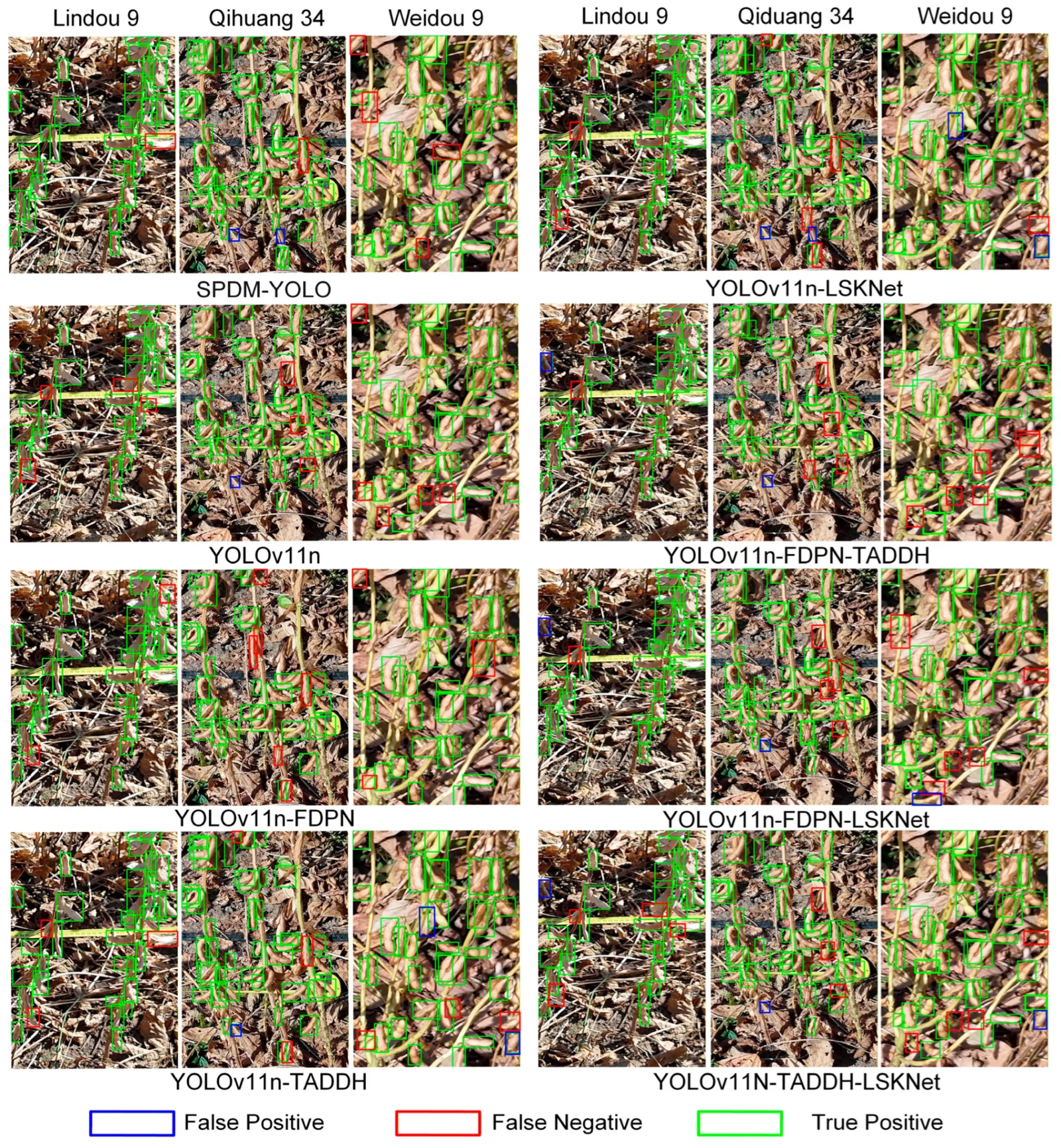
Comparison of detection performance in the ablation experiments. Green boxes indicate true positives, blue boxes indicate false positives, and red boxes indicate false negatives.

In Figure 8, the detection performance of all eight ablation variants is visually compared across three soybean cultivars (Lindou 9, Qihuang 34, and Weidou 9). Green boxes indicate true positive detections, blue boxes indicate false positives, and red boxes indicate false negatives.

From the baseline YOLOv11n to single-module, dual-module, and the full SPDM-YOLO model, the number of missed detections and false classifications decreases progressively. The full SPDM-YOLO achieves the most complete detection of densely clustered pods, with noticeably fewer false negatives in overlapping pod regions compared with the baseline and single-module variants.

Notably, all evaluated models exhibit the most stable recognition performance on Lindou 9, which has a relatively dispersed pod distribution and open branch architecture. Weidou 9, with its denser pod arrangement and more compact canopy structure, shows more missed detections across all variants, particularly in the baseline model. Across all cultivars, background stems or residual petioles are occasionally misclassified as pods, but such false positives are substantially reduced in the full SPDM-YOLO model due to its improved feature discrimination capability. Quantitative results of the ablation experiments are summarized in Table 1.

### 3.3. Experimental Results of Different Models

We trained the same dataset on YOLOv8n, YOLOv10n, YOLOv12n, and the proposed SPDM-YOLO model; the complete results are shown in Table 2. Among the baseline models, YOLOv12n achieved the lowest FLOPs (6.5 G) due to its attention-centric architecture, while YOLOv8n offered the fastest inference (134.6 FPS). The proposed SPDM-YOLO achieved the highest detection accuracy, with precision, recall, and mAP@0.5 reaching 88.2%, 87.0%, and 94.5%, respectively, while maintaining a comparable parameter count (2.78 M) to YOLOv10n and YOLOv12n. Although its inference speed (93.2 FPS) was lower than the baseline models, it remains sufficient for offline field phenotyping. For UAV deployment, the actual detection–tracking pipeline at 1280 × 768 video resolution achieved approximately 4 FPS using OC-SORT, which is adequate for post-flight analysis where each 6-m plot is captured in approximately 12 s at 0.5 m/s flight speed. Overall, SPDM-YOLO demonstrates robust pod detection capabilities and can be integrated with tracking algorithms for video-based pod counting.

### 3.4. Comparison and Selection of Multi-Object Tracking Algorithms for Video-Based | Pod Counting

To select the most suitable tracker for field soybean pod counting, three representative tracking algorithms (ByteTrack, BoT-SORT, and OC-SORT) were evaluated across all 27 experimental plots using the same SPDM-YOLO detector. The comparison focused on counting accuracy and inference speed.

As shown in Table 3, OC-SORT achieved the lowest dual-view fusion MAE (66.3 pods) and the highest R^2^ (0.741), outperforming ByteTrack (MAE = 86.1, R^2^ = 0.605) and BoT-SORT (MAE = 89.9, R^2^ = 0.485). Although ByteTrack offered the fastest inference (69.2 ms/f), its trajectory association was less stable under field occlusion and motion blur, leading to larger counting errors. BoT-SORT improved association via ReID but still underperformed OC-SORT in accuracy. OC-SORT’s observation-centric recovery mechanism maintained trajectory continuity during brief occlusion, yielding the most accurate and stable counts. Because field pod phenotyping in this study is performed as an offline post-flight analysis rather than real-time on-board inference, counting accuracy was prioritized over inference speed in tracker selection. OC-SORT’s inference speed (248.9 ms/f, approximately 4 FPS) is therefore acceptable: each 6-m plot video (approximately 12 s at 0.5 m/s flight speed) can be processed within one minute, sufficiently fast for high-throughput offline phenotyping. Based on these results, OC-SORT was selected as the tracking algorithm for all subsequent analyses.

### 3.5. Phenotypic Response of Soybean Pod Set Under Defoliation Treatments and Video-Based Pod Counting Results

Defoliation treatments, by establishing gradient levels of source limitation, not only alter soybean canopy architecture and light transmission characteristics but also directly regulate pod number, forming gradient phenotypic conditions that provide a test basis for systematically evaluating the counting robustness of PodCountNet under diverse canopy environments.

**Figure 9:**
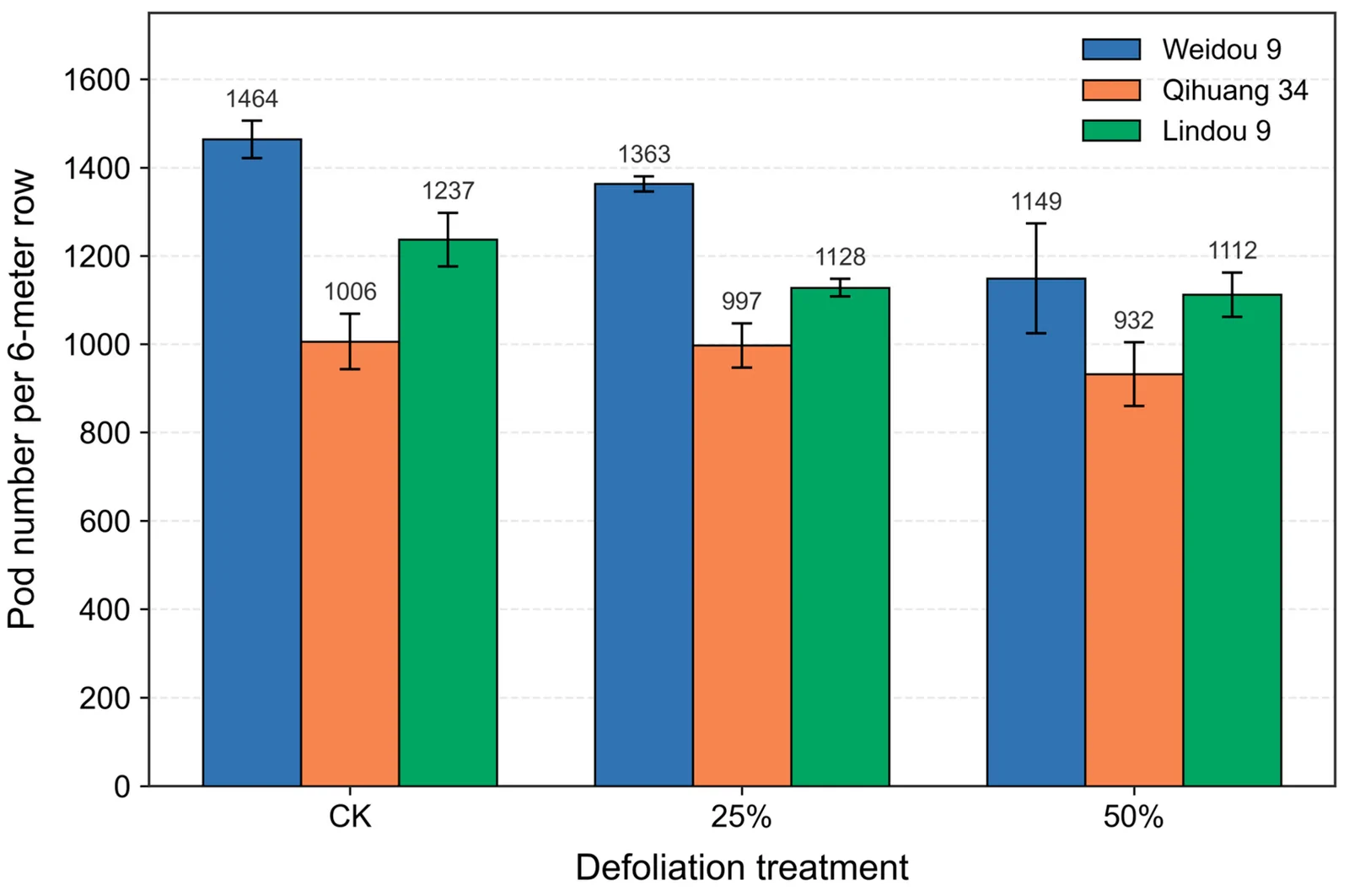
Pod number at maturity under different defoliation treatments for three soybean cultivars. Values are means ± SD of three biological replicates (*n* = 3).

In terms of manually measured pod set, the pod number of all three soybean cultivars showed an overall decreasing trend with increasing defoliation intensity, reflecting the regulatory effect of photosynthetic source limitation on soybean pod formation. Among them, Weidou 9 exhibited the most significant response of pod number to defoliation: the manual count was 1464 in the control group (CK), decreased to 1363 under 25% defoliation, and further dropped to 1149 under 50% severe defoliation, with an overall reduction of 21.5%. Qihuang 34 had the lowest baseline pod number, with 1006 pods in the CK group, a slight decrease to 997 under 25% defoliation, and 932 under 50% defoliation, showing a relatively gentle decline. The reduction in pod number of Lindou 9 was mainly concentrated in the moderate defoliation stage: 1237 pods in the CK group, decreased to 1128 under 25% defoliation, and only slightly dropped to 1112 under 50% severe defoliation, indicating that this cultivar has a stronger pod retention capacity under severe source limitation. The pod number responses across cultivars and defoliation treatments are summarized in Figure 9.

Using the selected OC-SORT tracker, video-based pod counting was performed for all 27 plots under both front-lit and back-lit conditions. Based on the video sequences of different cultivars and defoliation gradients described above, this study adopted the SPDM-YOLO detection model coupled with the OC-SORT multi-object tracking algorithm to perform pod tracking and counting. The counting performance under front-lit and back-lit observation conditions was compared using manual measurements as the ground truth. The complete quantitative results are summarized in Table 4.

Overall, the tracking counts under front-lit conditions showed higher consistency with the manual ground truth, with smaller and more stable counting deviations across all treatment groups. In contrast, back-lit conditions generally exacerbated counting fluctuations and amplified the magnitude of deviations, indicating that illumination condition is a core environmental factor affecting the stability of video-based pod counting.

Distinct differences in counting performance were observed among cultivars. Lindou 9 delivered the most stable performance under both lighting conditions, with absolute errors ranging from 49 to 130 under front-lit conditions and 57 to 147 under back-lit conditions, representing the lowest overall deviation level. This finding suggests that the pod morphology and dispersed pod distribution of Lindou 9 are more favorable for stable detection and tracking by the algorithm. In comparison, Weidou 9 was the most susceptible to lighting interference. Under back-lit conditions, the counting error of the 25% defoliation group increased sharply, with an absolute error reaching 528, reflecting a clear limitation in the robustness of single-view counting for dense-pod cultivars under complex lighting environments.

There was no simple linear correlation between defoliation ratio and counting error; instead, the effect of defoliation on counting accuracy exhibited a significant interaction with lighting conditions. Under front-lit conditions, the increase in defoliation ratio did not cause significant fluctuations in counting deviation, and the absolute errors of all treatments remained within a relatively stable range, meaning changes in canopy structure did not exert a notable impact on detection stability under front lighting.

Under back-lit conditions, the effect of defoliation degree on counting accuracy presented stage-specific characteristics. Moderate defoliation (25%) aggravated the heterogeneity of canopy shadows and significantly increased counting errors in some cultivars. For example, in the 25% defoliation group of Weidou 9, severe shadow occlusion led to massive missed detections, resulting in a tracking count of only 835, which was far lower than the manual ground truth of 1363. For Qihuang 34, the non-defoliated CK group showed the highest error under back-lit conditions, with a tracking count of 1286, 280 higher than the manual value of 1006. This was mainly attributed to the intertwined canopy branches and leaves in the non-defoliated state, which caused extensive false detections as pod contours were confused with branches under back lighting. When the defoliation ratio increased to 50%, reduced leaf occlusion alleviated missed detections caused by canopy overlap to some extent, and counting errors decreased accordingly in most groups. Specifically, the back-lit absolute error of Qihuang 34 under 50% defoliation dropped to 47, the lowest level among all treatments for this cultivar. However, severe defoliation was also accompanied by exposed branches and enhanced background interference, which limited the continuous decline of errors. For this reason, the error of cultivars such as Lindou 9 in the 50% defoliation group did not continue to decrease with increasing defoliation degree.

A two-way ANOVA was conducted on manual pod count with cultivar and defoliation treatment as fixed factors. Both cultivar (F_2,18_ = 68.06, *p* < 0.001) and defoliation treatment (F_2,18_ = 16.71, *p* < 0.001) had significant main effects, with a significant cultivar × treatment interaction (F_4,18_ = 3.75, *p* = 0.022). Post hoc comparisons indicated that the 50% defoliation treatment significantly reduced pod number compared with CK (*p* = 0.048), while the difference between CK and 25% defoliation was not statistically significant.

The above results demonstrate that single-view video pod counting is susceptible to the combined interference of illumination conditions, canopy structure and cultivar characteristics, and it is difficult to achieve stable and accurate pod quantification relying on a single observation perspective. Therefore, a dual-view fusion strategy was further adopted in this study to integrate counting results from front-lit and back-lit perspectives, so as to reduce the systematic deviation of single-view counting through complementary information.

### 3.6. Results of the Pod Count Prediction Regression Model

To achieve precise soybean yield prediction, this study employs the OC-SORT object tracking algorithm to dynamically count pods in soybean row videos captured by drones. During data acquisition, aerial footage of the same soybean row was captured from both front-lit and back-lit perspectives. Predicted pod counts were extracted from the corresponding videos, denoted as front-lit prediction (X_1_) and back-lit prediction (X_2_). Ground-truth pod counts (Y) obtained through manual sampling served as labels. A binary quadratic polynomial regression model was constructed to fit the prediction results, with its mathematical expression shown in Equation (7):

(7) $$Y=424.99+2.962X_{1}-2.768X_{2}+0.000484X_{1}^{2}-0.000742X_{1}X_{2}+0.001528X_{2}^{2}$$

**Figure 10:**
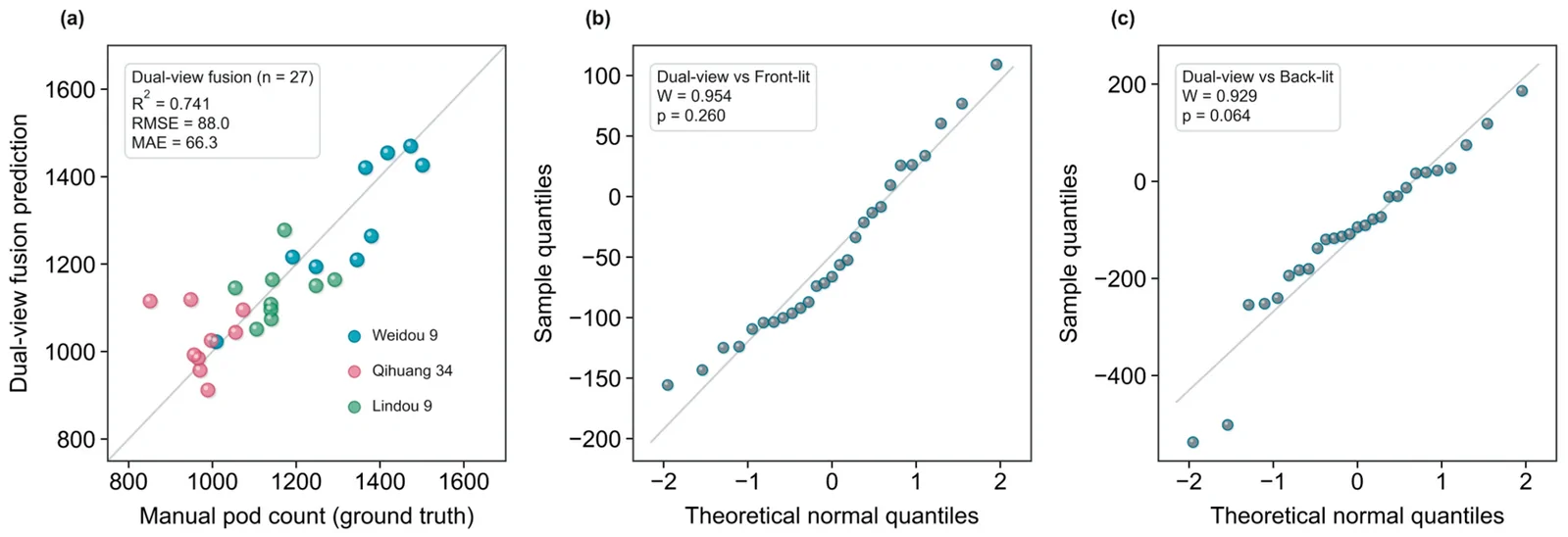
Evaluation of the dual-view fusion regression model (*n* = 27). (**a**) Observed versus predicted pod number; the gray solid line represents the 1:1 reference line (MAE = 66.3, RMSE = 88.0, R^2^ = 0.741). (**b**) Q-Q plot of the paired error difference between dual-view fusion and front-lit counting. (**c**) Q-Q plot of the paired error difference between dual-view fusion and back-lit counting.

The binary quadratic polynomial regression was fitted using all 27 individual plots, with front-lit and back-lit counts as independent variables and manual pod count as the dependent variable. At the individual plot level (*n* = 27), the dual-view fusion model achieved R^2^ = 0.741, MAE = 66.3 pods, and RMSE = 88.0 pods (Figure 10a). By comparison, single-view front-lit counting had MAE = 114.2 (RMSE = 136.2), and back-lit counting had MAE = 172.9 (RMSE = 233.0).

A paired t-test was used to compare the error reduction in dual-view fusion relative to single-view counting. The normality of the paired differences was first verified using the Shapiro–Wilk test. For the difference between dual-view fusion and front-lit counting errors, the test yielded W = 0.954, *p* = 0.260; for the difference between dual-view fusion and back-lit counting errors, W = 0.929, *p* = 0.064. Both *p*-values were above the 0.05 significance threshold, indicating that the normality assumption was satisfied and the paired t-test was statistically appropriate. The corresponding Q-Q plots are provided in Figure 10b,c for visual validation.

The paired t-test confirmed that dual-view fusion significantly reduced prediction error compared with both front-lit counting (t_26_ = −3.53, *p* = 0.002) and back-lit counting (t_26_ = −3.43, *p* = 0.002), corresponding to a 42.0% and 61.7% reduction in MAE, respectively. These results indicate that dual-view fusion provides reliable pod number estimation at the individual plot level and substantially outperforms single-view counting.

By integrating video counting results from both front-lit and back-lit perspectives, this approach overcomes errors caused by pod occlusion and lighting variations inherent to single-angle observations, achieving more stable and accurate yield predictions. These findings provide a reliable modeling foundation and methodological support for drone-based video-based pod number estimation at the field scale.

## 4. Discussion

### 4.1. Robust Pod Phenotyping Under Altered Photosynthetic Source Conditions

The PodCountNet framework proposed in this study is explicitly designed to support field-scale photosynthesis and source–sink research, rather than merely serving as an object detection algorithm. Compared with existing soybean pod detection methods that are mostly developed and validated under uniform laboratory or controlled conditions, our study conducted model training and validation entirely on UAV imagery from field-grown plants subjected to graded defoliation treatments. This experimental design intentionally introduced systematic variation in canopy structure, leaf occlusion, and light interception—all direct consequences of altered defoliation-induced source limitation—creating a more challenging and ecologically realistic test environment than controlled settings.

**Figure 11:**
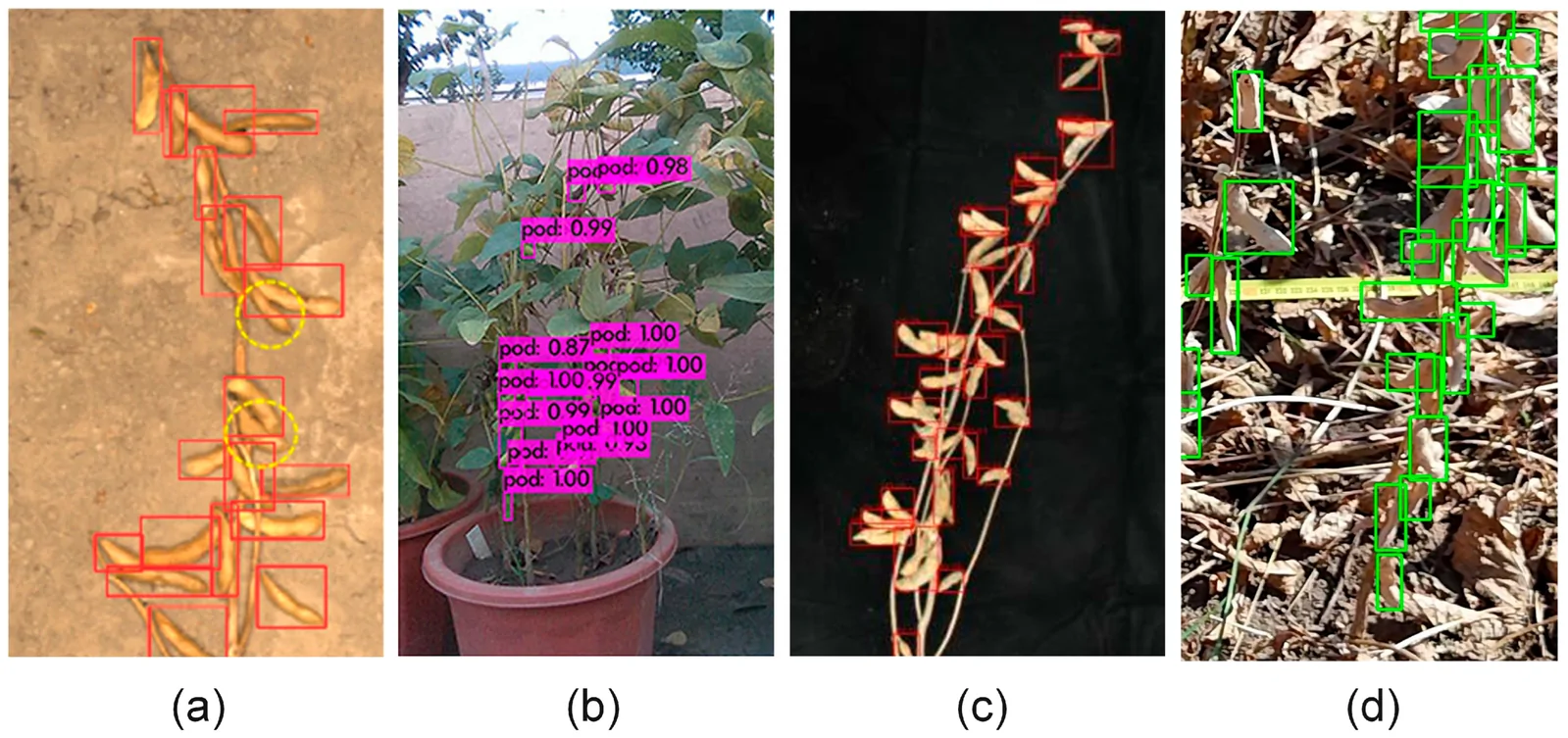
Visual comparison of pod detection results. (**a**) SmartPod; (**b**) Improved YOLOv3; (**c**) DARFP-SD + ResNet; (**d**) SPDM-YOLO.

As illustrated in Figure 11, existing pod detection studies were largely validated imagery captured under simplified scenarios: Figure 11a shows soybean plants against a smooth, clean soil background; Figure 11b was acquired under potted experimental conditions; Figure 11c depicts harvested whole plants photographed indoors against a black backdrop. In contrast, SPDM-YOLO was evaluated on imagery from real field canopies, as shown in Figure 11d. Data from controlled settings offer clear target features, minimal background interference, and uniform illumination, which facilitate feature extraction and model training, and enable high reported accuracy under ideal conditions. As summarized in Table 5, established methods such as SmartPod, Improved YOLOv3, and DARFP-SD + ResNet all report precision and recall above 85% under such ideal shooting conditions with clean backgrounds and minimal occlusion. These results demonstrate the effectiveness of these methods within their respective validated scenarios. However, because field canopies exhibit substantially greater heterogeneity in leaf coverage, pod distribution, illumination, and background clutter than controlled settings, the direct transferability of these models to unconstrained field environments remains an open question that has not been systematically evaluated in the original studies.

In contrast, SPDM-YOLO achieved 88.2% precision and 94.5% mAP@0.5 across all defoliation gradients and cultivars, demonstrating its applicability for pod detection under complex field canopy conditions induced by photosynthetic source reduction. This field-relevant performance arises from both algorithmic optimization and ecologically relevant data collection. By embedding LSKNet for dynamic multi-scale receptive field adjustment and FDPN for enhanced small-target feature fusion, the model adapts to the large variation in pod appearance caused by differing canopy depths and occlusion levels. More importantly, our dataset spans multiple cultivars and defoliation intensities, capturing the range of phenotypic variation encountered in source–sink field trials. This ensures that the framework has been validated not only under optimal growth conditions, but also under the altered canopy states that are central to photosynthesis research, For quantitative comparison, SmartPod achieved 91.5% precision and 90.1% recall on relatively clean field plants with minimal occlusion [15], while Improved YOLOv3 reached 90.3% precision and 87.6% recall on harvested plant images against a uniform black background [30]. Our SPDM-YOLO achieves 88.2% precision across diverse defoliated canopies with varying occlusion and illumination, which is highly comparable to these controlled-environment results despite the substantially more challenging imaging conditions. thereby extending the practical scope of soybean pod phenotyping from controlled environments to real-world field production settings.

### 4.2. Phenotypic Variation in Sink Traits Under Different Source Limitations

All defoliation treatments were manually applied at the R2 full flowering stage to impose graded source limitation during reproductive development. UAV imaging and pod detection were conducted at the maturity stage, when soybean leaves had naturally abscised across all treatments. At this growth stage, canopy structure was composed solely of stems, branches and pods, and the phenotypic differences among treatments—including pod number, pod spatial distribution and branch architecture—were the lasting outcomes of source limitation imposed at flowering. The approximately 10-week interval between R2 defoliation and maturity-stage imaging ensured that the observed pod-number differences reflected physiological responses to altered source strength (flower abortion, pod set, and retention), rather than transient physical effects of leaf removal. Accordingly, the divergent counting performance across defoliation treatments and cultivars reflects the real interactions between defoliation-induced source limitation (manipulated at flowering), resultant canopy architectural traits at maturity, and phenotyping reliability during sink formation.

As shown in Table 4, no simple linear correlation existed between defoliation ratio and counting error. Given the absence of leaf tissue at maturity, the variation in counting accuracy may be jointly attributed to three interrelated canopy attributes shaped by flowering-stage defoliation and genotypic characteristics: the degree of pod-to-pod and branch-to-pod occlusion, the heterogeneity of shadow distribution within the leafless canopy, and the overall exposure level of individual pods. It should be noted that these structural factors were not directly quantified in the present study, and the following interpretation is therefore presented as a hypothesis-based explanation rather than a statistically verified causal relationship.

**Figure 12:**
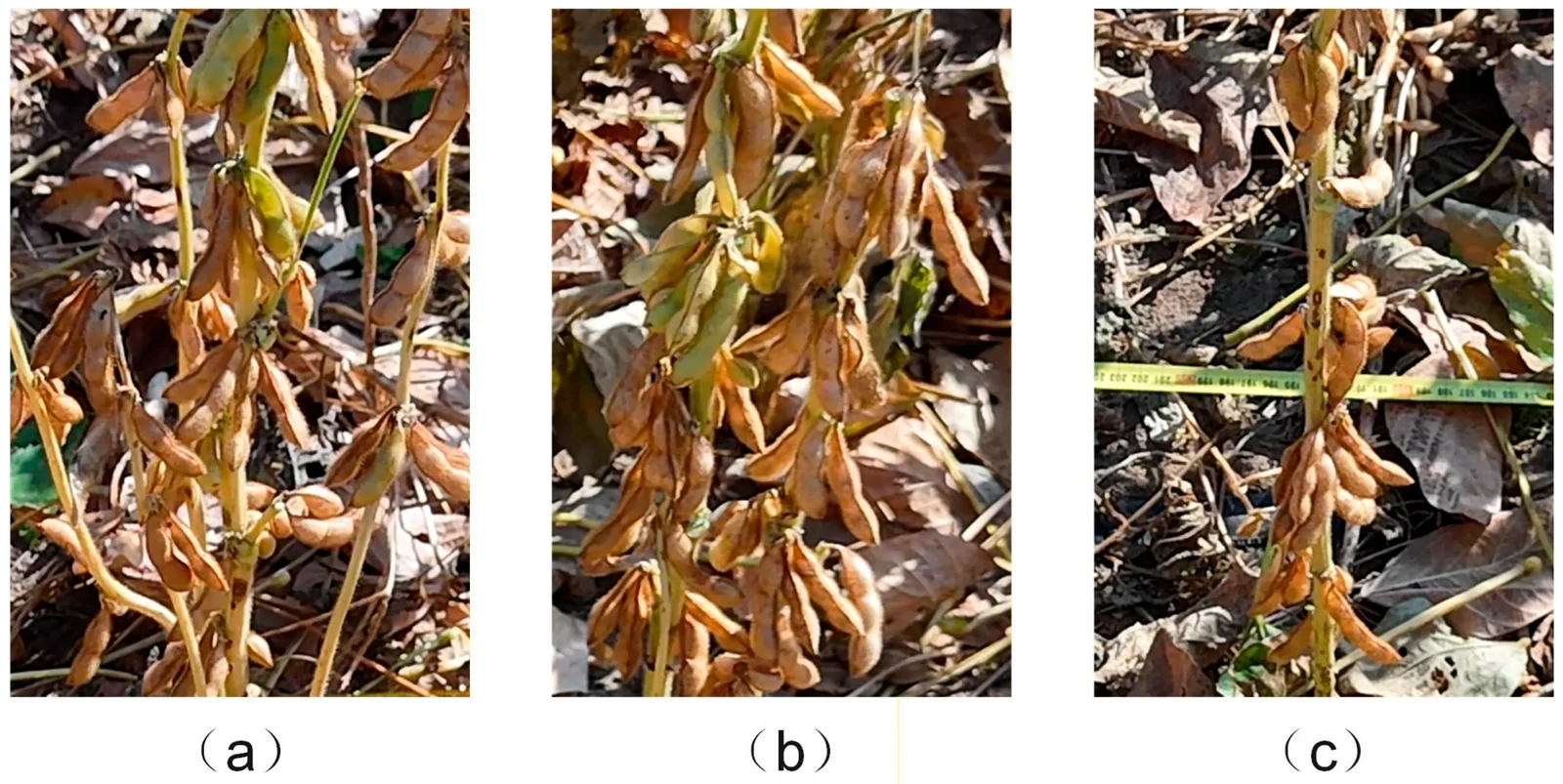
Representative back-lit canopy images of Weidou 9 across three defoliation levels. (**a**) CK: dense pod arrangement and interwoven branches with relatively uniform shadows; (**b**) 25% defoliation: fragmented dappled shadows and persistent pod-to-branch occlusion, corresponding to the highest counting error among all treatments; (**c**) 50% defoliation: sparser pod distribution and more uniform canopy shadows, corresponding to reduced counting errors.

Representative back-lit canopy images of Weidou 9 (Figure 12) reveal clear qualitative differences in pod density, branch occlusion, and shadow heterogeneity across the three defoliation levels, which are consistent with the observed error patterns.

Under front-lit conditions, uniform incident illumination reduces shadow contrast within the canopy and weakens the interference from uneven shadow distribution. Even under dense pod arrangement and branch occlusion, the algorithm maintains stable feature extraction capability. As a result, counting errors remained relatively stable across all defoliation gradients for all three cultivars, with absolute errors fluctuating within a narrow range. This indicates that under sufficient illumination, the framework retains robust detection performance regardless of differences in pod density and canopy architecture caused by flowering-stage source limitation.

Under back-lit conditions, by contrast, the three structural attributes exert a more pronounced combined effect, leading to stage-specific error patterns along the defoliation gradient. In the 25% defoliation group, moderate source limitation resulted in a partially altered canopy structure: pod number was only slightly reduced compared with the control, but branch and pod distribution became more uneven. This fragmented canopy architecture created highly heterogeneous dappled shadows under back lighting, while pod-to-pod and branch-to-pod occlusion remained substantial. The superposition of intensified shadow heterogeneity and persistent occlusion severely disrupted pod feature extraction, which is consistent with the sharp surge in counting errors for dense-pod cultivars such as Weidou 9: its back-lit absolute error jumped from 94 in the CK group to 528 in the 25% defoliation group, with massive missed detections triggered by severe shadow and occlusion interference.

When defoliation intensity increased to 50%, stronger source limitation led to a marked reduction in pod number and a more sparse pod distribution at maturity. On the one hand, this alleviated pod-to-pod overlap and branch occlusion; on the other hand, the overall canopy shadow pattern became more uniform rather than locally fragmented, and individual pod exposure was significantly improved. These joint changes drove down counting errors in most treatment groups. For instance, the back-lit absolute error of Weidou 9 dropped from 528 at 25% defoliation to 87 at 50% defoliation, and that of Qihuang 34 decreased from 173 to 47, reaching the lowest level among all its treatments. It is notable that for Qihuang 34, the highest back-lit error appeared in the non-defoliated CK group (absolute error of 280). In this group, intact source capacity supported full pod set and dense branch architecture at maturity; under back lighting, the intertwined branches and densely arranged pods caused severe target confusion and extensive false detections—a result that also reflects the combined influence of occlusion and shadow interference under high pod-density canopy conditions.

Cultivar-specific performance differences further support the roles of pod exposure and canopy structural traits. Lindou 9 exhibited the most stable counting performance under both lighting conditions, which corresponds to its inherently dispersed pod distribution and relatively open branch architecture. These traits reduce the probability of pod-to-pod and branch-to-pod occlusion, maintain relatively stable pod exposure under different source limitation levels, and thus improve phenotyping reliability. In contrast, the dense pod arrangement and compact branch canopy of Weidou 9 result in lower pod exposure and higher sensitivity to occlusion and lighting interference, leading to larger performance fluctuations under adverse observation conditions. This genotypic variation further emphasizes that robust field phenotyping tools must accommodate the diverse canopy architecture and sink trait characteristics present across soybean germplasm, to ensure broad applicability in source–sink physiology research and breeding applications.

These findings collectively demonstrate that single-view pod counting is highly sensitive to canopy structural changes induced by flowering-stage source strength variation, even in the leafless maturity stage. Its error pattern may reflect the integrated influence of pod occlusion degree, canopy shadow heterogeneity and pod exposure level shaped by both treatments and genotypes. Future studies incorporating quantitative measurements of canopy light distribution, occlusion fraction, and pod exposure angle via 3D point cloud scanning or radiative transfer modeling would enable more rigorous validation of these structural drivers. This further highlights the necessity of multi-view fusion to achieve reliable phenotyping across diverse photosynthetic source environments and genotypic backgrounds.

Biologically, the reduction in pod number from 1464 (CK) to 1149 (50% defoliation) in Weidou 9 is consistent with the cumulative impact of flowering-stage defoliation-induced source limitation on pod set and retention. This pattern aligns with the well-documented source–sink regulation in soybean: defoliation during reproductive stages generally reduces pod set and retention, and the magnitude of pod loss increases as defoliation timing approaches the critical pod-set window, with significant genotypic variation across cultivars [7]. The 21.5% pod reduction observed in Weidou 9 under 50% flowering-stage defoliation is consistent with the expectation that source limitation during early reproductive development imposes strong constraints on sink formation. We hypothesize that reduced assimilate supply during early reproductive development may affect both pod initiation and pod abortion, potentially mediated through changes in canopy radiation interception, plant hormonal balance, and source–sink assimilate allocation. However, these physiological pathways were not directly measured in the present study, and further dedicated experiments are needed to verify the specific mechanisms underlying the observed pod number responses.

### 4.3. Innovative and Practical Video-Based Soybean Pod Counting Method for Complex Field Backgrounds

A core innovation of this study is that it advances pod phenotyping from static image detection to continuous video-based quantification, thereby enabling the high-throughput required for large-scale photosynthesis and source–sink field trials. Most existing pod phenotyping methods rely on detached plants or static ground-based imaging, which are destructive, low-throughput, and unsuitable for monitoring dynamic sink development across entire field experiments.

By integrating OC-SORT multi-object tracking with SPDM-YOLO, PodCountNet achieves continuous, non-destructive pod counting along entire soybean rows from UAV video. The tracking algorithm associates individual pods across consecutive frames using trajectory consistency, effectively avoiding duplicate counting and missed detections caused by UAV motion and transient occlusion. Among the three trackers evaluated, OC-SORT provided the most stable trajectory association under field occlusion, which directly translated to the lowest counting error. This video-based workflow dramatically increases phenotyping efficiency: a 6-m plot can be quantified in seconds, compared with tens of minutes required for manual counting. This throughput makes it feasible to phenotype hundreds of plots in a single day, which is essential for evaluating source–sink responses across large breeding populations or multi-factor field experiments. The accuracy advantage of multi-view fusion for pod counting has also been verified in other field phenotyping systems. Using ground-based stereoscopic imaging of soybean harvest stocks, Liu and Tang reported that dual-view imaging reduced the mean absolute percentage error of pod counting from 20.12% (single view) to 11.91% by mitigating occlusion errors [32]. In this study, the dual-view fusion strategy based on front-lit and back-lit UAV video achieved 42.0% and 61.7% MAE reduction relative to single-view counting, further extending the application scenario of multi-view pod phenotyping from ground platforms to low-altitude UAV systems.

From a photosynthesis research perspective, this framework addresses an important phenotyping challenge. Previously, researchers could only estimate source activity indirectly through spectral indices, and could not directly measure sink traits at scale. PodCountNet enables direct, rapid quantification of pod number—the most important sink trait—across variable source-strength conditions. When combined with canopy spectral measurements and physiological sampling, this tool will allow researchers to quantitatively link photosynthetic source capacity, assimilate allocation, and sink development under field conditions. It also provides a practical platform for screening soybean germplasm with superior source–sink coordination and improved photosynthetic productivity.

While ground-based phenotyping platforms can offer finer spatial resolution, their wheeled chassis may cause soil compaction and plant damage in narrow-row layouts, and their throughput is substantially lower than UAV-based surveys. The two approaches are complementary rather than mutually exclusive.

### 4.4. Limitations and Future Work

This study has several limitations that will be addressed in future research:

(1) The current framework was validated at a single experimental site in Shandong Province. Its generalization performance across different climatic regions, soil types and planting patterns requires further verification.

(2) The model is currently optimized for 1-m ultra-low-altitude UAV imaging. Future work will optimize the feature extraction network to adapt to higher-altitude imaging for rapid, large-scale field yield estimation.

(3) Detection accuracy under back-lit conditions still has room for improvement. Low-light image enhancement algorithms will be integrated to further enhance environmental adaptability across variable canopy illumination conditions.

(4) The structural drivers of counting error variation—pod occlusion degree, canopy shadow heterogeneity, and pod exposure level—were not quantitatively verified in this study, and the current interpretation is based on qualitative canopy observation and empirical inference. Future studies will introduce terrestrial LiDAR 3D point cloud scanning or radiative transfer modeling to quantitatively decompose the independent contribution of each structural factor to counting errors.

(5) Currently the framework only quantifies pod number. Future research will integrate pod size and seeds per pod to improve yield estimation accuracy.

(6) Most importantly, future studies will integrate pod counting with physiological indicators such as chlorophyll fluorescence, canopy photosynthetic rate, and carbon assimilation measurements to establish a comprehensive high-throughput phenotyping platform for photosynthetic efficiency and source–sink.

## 5. Conclusions

This study addresses the phenotyping bottleneck for evaluating source–sink responses under variable photosynthetic conditions by proposing PodCountNet, an integrated detection–tracking framework for high-throughput soybean pod quantification based on UAV video imagery. The main findings are as follows:

(1) An improved SPDM-YOLO pod detection model was developed based on YOLOv11n. By incorporating LSKNet dynamic receptive field adjustment, a feature-focused diffusion pyramid network (FDPN), and a task-aligned dynamic detection head (TADDH), the model achieves strong detection performance for small, densely distributed pods across diverse canopy structures shaped by graded defoliation. It achieved 88.2% precision and 94.5% mAP@0.5 under complex field conditions, outperforming mainstream YOLO variants (YOLOv8n, YOLOv10n, and YOLOv12n) while maintaining a comparable parameter count (2.78 M).

(2) Integration of the OC-SORT multi-object tracking algorithm enabled continuous video-based pod counting, effectively reducing counting errors caused by pod occlusion, canopy structural variation and camera motion. Compared with ByteTrack and BoT-SORT, OC-SORT consistently achieved the lowest counting error across all 27 experimental plots and was therefore selected as the primary tracker for offline field pod phenotyping. This advance extends pod phenotyping from static single-frame detection to efficient continuous video quantification under variable field canopy conditions.

(3) A dual-view data fusion strategy combining front-lit and back-lit video counts yielded a binary quadratic polynomial regression model that achieved MAE = 66.3 pods, RMSE = 88.0 pods, and R^2^ = 0.741 at the individual plot level (*n* = 27), significantly outperforming single-view front-lit and back-lit counting (paired *t*-test, *p* < 0.01) and providing reliable pod number estimation across variable photosynthetic source conditions.

(4) The R2 full-flowering defoliation experiment revealed coordinated biological responses and phenotyping performance patterns. Biologically, pod number at maturity decreased consistently with escalating defoliation intensity across all three tested soybean cultivars, suggesting that flowering-stage defoliation-induced source limitation influences final pod retention and sink formation. Two-way ANOVA confirmed significant main effects of cultivar (F_2,18_ = 68.06, *p* < 0.001) and defoliation treatment (F_2,18_ = 16.71, *p* < 0.001), with a significant cultivar × treatment interaction (F_4,18_ = 3.75, *p* = 0.022), confirming that flowering-stage defoliation-induced source limitation influences final pod retention and sink formation. Methodologically, counting accuracy exhibited no simple linear correlation with defoliation ratio; instead, it was governed by the combined effects of inter-pod and branch-pod occlusion, canopy shadow heterogeneity, and individual pod exposure—all structural outcomes of source limitation and genotypic traits—with strong interactions with illumination conditions. Front-lit counting retained stable accuracy across all defoliation gradients, whereas back-lit counting was more susceptible to canopy structural variation: moderate defoliation (25%) induced the most drastic error increase in dense-pod cultivars due to fragmented shadow distribution and persistent occlusion, while severe defoliation (50%) partially alleviated occlusion and reduced counting errors in most groups. Comprehensive validation across three cultivars and three defoliation levels confirmed that PodCountNet delivers stable performance across divergent canopy architectures and source-strength gradients for field-based source–sink physiological research.

Beyond algorithmic advances, this study demonstrates that UAV-based pod counting can serve as an efficient high-throughput phenotyping strategy for quantifying sink responses under different defoliation-induced source limitations. The proposed framework provides a practical tool for investigating source–sink relationships, evaluating photosynthetic productivity, and supporting breeding strategies aimed at improving crop yield through enhanced photosynthetic efficiency.

## Figures and Tables

**Table 1:** Results of ablation experiments.

Model	P (%)	R (%)	mAP@0.5 (%)
SPDM-YOLO	88.2	87.0	94.5
YOLOv11n	86.0	85.5	90.4
YOLOv11n-FDPN	87.1	87.0	93.9
YOLOv11n-TADDH	87.9	86.0	93.5
YOLOv11n-LSKNet	88.2	86.5	94.3
YOLOv11n-FDPN-TADDH	87.8	86.8	94.0
YOLOv11n-FDPN-LSKNet	88.1	86.9	94.3
YOLOv11n-TADDH-LSKNet	88.0	**86.6**	94.1

**Table 2:** Performance comparison of different mainstream YOLO models. Params and FLOPs calculated at 640 × 640 input resolution. FPS measured on an NVIDIA RTX 3080 GPU with 640 × 640 input.

Model	P (%)	R (%)	mAP@0.5 (%)	Params (M)	FLOPs (G)	FPS
SPDM-YOLO	**88.2**	**87.0**	**94.5**	2.78	7.9	93.2
YOLOv8n	87.2	85.6	92.9	2.72	7.1	134.6
YOLOv10n	85.8	86.1	93.2	2.78	8.7	116.7
YOLOv12n	86.5	86.0	92.4	2.66	6.5	107.2

**Table 3:** Performance comparison of tracking algorithms across 27 plots.

Tracker	Dual-View MAE	Dual-View R^2^	Inference (ms/f)
OC-SORT	**66.3**	**0.741**	248.9
BoT-SORT	89.9	0.485	137.5
ByteTrack	86.1	0.605	**69.2**

**Table 4:** Counting results of the tracking algorithm under different soybean varieties and defoliation treatments. Values are means ± SD of three biological replicates.

Variety	Treatment	Manual Count	Front-Lit Tracking	Back-Lit Tracking	Front-Lit Absolute Error	Back-Lit Absolute Error
Weidou 9	CK	1464 ± 42	1530 ± 81	1558 ± 10	66	94
	25%	1363 ± 17	1196 ± 75	835 ± 117	167	528
	50%	1149 ± 124	1259 ± 119	1236 ± 91	110	87
Qihuang 34	CK	1006 ± 63	1172 ± 52	1286 ± 14	166	280
	25%	997 ± 50	1094 ± 41	1170 ± 69	97	173
	50%	932 ± 72	1058 ± 61	979 ± 55	126	47
Lindou 9	CK	1237 ± 61	1314 ± 76	1294 ± 74	77	57
	25%	1128 ± 20	1177 ± 27	1252 ± 36	49	124
	50%	1112 ± 50	1242 ± 34	1259 ± 45	130	147

**Table 5:** Identification Performance of Existing Soybean Pod Detection Models.

Method	P (%)	R (%)	References
SmartPod	91.50	90.10	[15]
Improved YOLOv3	90.30	87.60	[30]
DARFP-SD + ResNet	90.35	85.59	[31]

## Data Availability

The data presented in this study are available on request from the corresponding author.

## Abbreviations

The following abbreviations are used in this manuscript:

UAV	Unmanned Aerial Vehicle
mAP	mean Average Precision
MAE	Mean Absolute Error
FDPN	Feature-focused Diffusion Pyramid Network
TADDH	Task-Aligned Dynamic Detection Head
OC-SORT	Observation-Centric Simple Online and Realtime Tracking
IoU	Intersection over Union
